# CapEs: designing and implementing a *Cap*acity *Es*timator tool to support primary care workforce planners in Canada

**DOI:** 10.1186/s12913-026-15311-6

**Published:** 2026-08-07

**Authors:** Morgan Price, Tlell Elviss, Elka Humphrys

**Affiliations:** 1 Faculty of Medicine, Department of Family Practice, University of British Columbia, Vancouver, BC Canada; 2 Island Medical Program, Faculty of Medicine, University of British Columbia, Victoria, BC Canada

**Keywords:** Primary care, Health human resources, Primary care workforce planning, Knowledge translation, Complex intervention, Simulation modeling, Simulation dynamics, Team-based primary care

## Abstract

**Background:**

Primary care is a cornerstone of high-quality healthcare systems and primary care workforce planning requires more robust tools than panel size targets, particularly as jurisdictions shift to team-based care. Few such tools exist in Canada. This paper describes the design of *CapEs*, a primary care CAPacity EStimator simulation software and its formative use, applied in facilitated workshops for primary care planners assessing primary care need and workforce capacity.

**Methods:**

CapEs was developed through a partnership with the British Columbia (BC) Ministry of Health. This paper describes the intervention development study: requirements were gathered through stakeholder interviews and a co-design workshop. The simulation model was built in Stella Architect 3, informed by published literature and additional evidence from seven modified Delphi studies. CapEs was implemented by embedding it in facilitated workshops where the research team used the tool in real-time with workforce planners to explore community-level workforce plans and team configurations. Outputs were compared to current BC panel size targets.

**Results:**

Requirements led to the design of a simulation model that could be used with limited local data to predict the percentage of primary care needs met by clinically diverse primary care teams. CapEs was implemented as a complex intervention: the simulation model was embedded in workshops where the authors used CapEs with workforce planners to explore primary care plans, discussing implications of the model’s predictions. Forty-five plans or communities have been reviewed with CapEs in three provinces in Canada. The facilitated workshop format allowed planners to explore scenarios in real-time while the research team provided clinical context and navigated data limitations. Benchmarking against BC policy panel targets showed broadly consistent results with some differences that suggest refinements to both the model and policy targets.

**Conclusions:**

Tools like CapEs that estimate capacity match offer a more nuanced view of primary care workforce adequacy compared to panel targets. Embedding such tools in facilitated workshops made them accessible to planners with varying knowledge and data availability. Further validation and population-level data are needed to strengthen these models for primary care workforce planning in Canada.

**Supplementary Information:**

The online version contains supplementary material available at https://doi.org/10.1186/s12913-026-15311-6.

## CapEs software paper: Background

### Primary care capacity

Primary care is the cornerstone of high quality, sustainable healthcare systems [1]. It is associated with reductions in mortality, hospitalizations, and costs, while improving access to care, illness prevention, and population health [2–4]. Canada is experiencing a primary care workforce (PCW) crisis [5–8] with 22% of Canadians being unattached (they do not have a regular primary care provider) [9–11]. Reasons relate to care demand (population size, complexity) and workforce supply [12–19].

Interdisciplinary primary care teams (primary care provider with at least one other clinical professional sharing care responsibility for a population of patients, or panel) are an established and internationally recognized key part of effective primary care [6, 8, 14, 20–25]. Teams can increase capacity [26, 27], enhance quality [28, 29], increase resilience [30–32], and improve patient experience [33]. Teams are also a recruitment strategy [34, 35]. Several Canadian provinces have implemented team-based care (TBC) [36] and are optimizing to meet populations’ needs [37–39]; however, the workforce planning formulas used may be overly simplified. There are few tools to support detailed capacity estimating for workforce planning.

Evidence-based workforce planning strategies are required that factor in team design and patient complexity [40] to better ensure that primary care meets clinical need [8, 41–43]. Reliance on simple historical or population trends often results in suboptimal service delivery outcomes. Better tools are needed to support PCW planners as they create policies to improve capacity in primary care.

### Capacity estimation in primary care

Despite challenges defining optimal panel sizes [44], PCW planners must use some method for workforce planning. British Columbia’s (BC) strategy defines panel size targets for 3 broad practice categories (urban, rural, and vulnerable population) with different targets for different providers (Table 1). These targets are also scaled for community-level primary care workforce plans [45].

**Table 1:** BC attachment targets (number of patients / provider or panel size) for different providers and populations

Provider Type	Urban Panel	Rural/Remote	Vulnerable Population
Family Physician (FP)	1250	800	650–800
Nurse Practitioner (NP)*	1000	800	600–700
Registered Nurse (RN)**	+ 500	+ 400	NA
Allied Health	NA	NA	1 full time equivalent (FTE) per 200–400 unattached people

* Nurse Practitioners are PCPs in BC

** Registered Nurse panel numbers would be added to the PCP panel target [45].

Studies estimating capacity fall into two categories: First, measurement of capacity from actual data. Second, theoretical studies that predict demand and/or capacity. Both types of studies have challenges: Actual data will underestimate need [46, 47] as systems are typically capacity constrained, so unmet need is not captured in the observed data. Theoretical studies may over index some factors and miss others.

Several studies have examined the theoretical primary care need. Altschuler et al. in 2012 [48] reviewed clinical practice guidelines in the United States and predicted the total clinician time required for a population and then proposed various panel sizes (from 1387 to 1947 patients) based on team configurations. This study was revisited by Porter in 2023 to provide a refined model of primary care demand [49]. Ozen and Balasubramanian developed an excel based tool to propose optimal panel sizes that used multimorbidity case-mix and calculated “overflow” (where demand exceeds capacity) when optimizing panel sizes [40].

In Canada, one study in Ontario measured mean annual primary care visits with chronic conditions, which ranged from approximately 3 for patients with no chronic conditions to 10 for those with 6 or more chronic conditions [50]. In BC the average number of primary care visits in 2017-18 was 3.6–3.9 visits / year depending on rurality [51]. Primary care provider (PCP) visits rates have declined in BC [52] potentially due to a need for longer appointments to address an aging population with multi-morbidity and higher acuity.

Although these studies provide evidence that would be helpful for planners, the findings are difficult for planners to use directly, and they default to coarse estimates like simple panel size targets. Tools are therefore needed to translate evidence into locally relevant estimates for primary care workforce planners.

### Paper contribution

In this intervention development study, we describe the design of *CapEs*, a primary care *Cap*acity *Es*timator simulation, and a formative evaluation of its use within a facilitated *CapEs workshop* that helps PCW planners weigh care needs against the capacity of team configurations when reviewing practice- and community-level primary care workforce plans.

## Methods

This intervention development study focused on the iterative design and use of the CapEs simulator to support primary care workforce planning in BC, Canada. CapEs was requested and supported through a partnership with the BC Ministry of Health. Requirements work began in 2022 with several iterations of CapEs that year. A related study (CIHR FRN 169130) expanded the evidence base for CapEs through 7 modified Delphi studies and supplied needed input parameters for CapEs (described below, paper under review). For more information on Delphi, see Niederberger [53]. 

### Design and development of CapEs

#### CapEs v1 (prior work)

Prior to the request from the Ministry of Health, in 2019, the researchers independently tested an initial version 1 prototype (using Apple Numbers spreadsheet), built based on existing literature. This version was not used with stakeholders, but the literature and work informed the new model described here.

### User requirements

The requirements development had four steps:

1. **Stakeholder Interviews**: semi-structured requirements gathering interviews were completed with primary care leader team in the BC Ministry of Health (the group responsible for reviewing workforce plans).
2. **Requirements Synthesis**: interview notes were reviewed and requirements extracted into a subset of concepts that were prepared for further exploration and validation.
3. **Requirements Workshop**: 2-hour co-design workshop with the BC primary care leadership team, confirming direction using “even-over” statements (a prioritization method in which participants choose what they value even over another desirable option, forcing explicit trade-offs) [54] and exploring target end users.
4. **Literature Search / Policy Review**: Based on user stakeholder requirements, we searched for available evidence related to health workforce planning to inform the simulation. Policy documents provided by stakeholders were also reviewed to compare with CapEs.

### Building the CapEs simulation

CapEs was developed in Stella Architect 3.x, which is a rapid visual design environment that includes features for simulation modeling and user interfaces (www.iseesystems.com). Theoretical foundations for CapEs came from the Altschuler paper [48] and the Porter paper [49]. These papers provided a useful way to break down primary care into categories of acute, chronic, and prevention care. Second, they provided an estimate of what could be delegated to a primary care team from a primary care provider for each category. These were building blocks for the simulation. Early versions were used with end users in workshops and ongoing feedback allowed for refinement of the design, seeing where health planners need more information and what data may or may not be available to inform the model.

### Formative evaluation of CapEs

#### Use: CapEs workshops

We incorporated CapEs into a facilitated workshop with PCW planners. A specific clinic or community plans would be reviewed in the workshop. Before a CapEs workshop, the researchers would review available data (population size, characteristics and provider FTE counts). The researchers facilitated a discussion, using CapEs in real-time to show different assumptions and configurations of the population or the team. Snapshots of the different configurations were recorded, and a short report was provided back to the PCW planners for their use.

#### Feedback & benchmarking

During the design of CapEs, feedback from PCW planners was gathered through informal field notes and post-workshop discussions and this guided further CapEs feature design through the iterative process. CapEs models were benchmarked against the panel sizes proposed in BC primary care strategy (Table 1). The CapEs outputs were then used to check for consistency between versions.

Requirements gathering and application of CapEs were part of Learning Cycle projects and thus considered quality improvement where human ethics and consent to Participate declarations were considered not applicable.

## Results

### Development results

#### Initial user requirements

The seven people in the BC primary care leadership team were individually interviewed by one member of the research team (MP); all were primary care executives/leads with knowledge of PCW planning in the BC Ministry of Health. Interview results were then analyzed by two members of the research team (MP, TE) to create a set of ten initial requirement choices to frame the workshop. Five of the seven members of the leadership team participated in the requirements workshop. The ten requirements were brought to the workshop and presented through the even-over statements to understand different perspectives. 60% of the requirements had 80% agreement (Table 2). The workshop was facilitated, and results analyzed, by two members of the research team (MP, TE).

**Table 2:** Requirements workshop outputs: six even-over statements where > = 80% ( > = 4/5) participants agreed on the priority

Preferred requirement	Chosen over this requirement
Have many levers to adjust	Simple application requiring little training
Match patient needs to team configuration	Considering only workforce availability
Support community level or team level service planning	Inform policy planning (e.g., provincial)
Focus on higher quality estimates for fewer roles	Having many roles included with lower quality estimates
Support modeling different primary care clinic types (urgent care only, longitudinal practice, small teams, larger teams)	Modeling specific domains of care (e.g. mental health and substance use, maternity care)
Modeling focused on clinical needs (e.g., urgent care, chronic care, prevention)	Modeling broader patient needs (e.g., related to social determinants of health)

There was also a very clear direction (100% agreement) on two expected user types for CapEs: clinic level leadership (e.g., clinic manager) and regional directors who review community plans. These became the personas for designing the CapEs experience.

### CapEs design

All iterations of the CapEs simulation model follow the same general visit design. CapEs predicts the average annual visit *need* of a population (e.g., a panel of patients in a clinic, or the population served within a network of teams) divided into acute, chronic, and prevention visits. It also calculates the visit *capacity* of primary care serving that population (e.g., FTEs of different roles). CapEs then simulates a year of care, predicting met and unmet need for each week in the simulation year. After early testing, a key design decision was to predict the care need met/unmet instead of predicting optimal FTE requirements for the team. An optimal-FTE output would imply that there could be an optimal team, a precision the available evidence and data cannot support, and would give planners a single answer. Predicting met and unmet need instead lets planners compare team configurations and reason through what-if scenarios to explore options based on funding or clinician availability predictions. This guided the design of the underlying model, the user interface, and the use of CapEs. Features were added based on user feedback from the CapEs workshops.

The current system dynamics model for CapEs (v3) is divided into three parts:

1. **The Population Model** calculates the visit need for the population. It can be adjusted for population size, complexity, prevention target, and rurality. Complexity adjusts the per capita visit demand. The slider is adjusted manually, based on human interpretation of available population level data like age pyramids, chronic-disease burden, metrics describing social determinants of health, or clinic mandates (e.g., for new clinics focused on populations). CapEs currently does not import patient level data to model visit demand.
2. **The Team Model** calculates the overall visit capacity of the team based on in-clinic FTEs of different clinicians. The user can also adjust the amount of shared care to simulate teams that are working higher or lower in their scopes of practice.
3. **Met and Unmet Need**: Care visits (patients’ needs being addressed) are modeled as the simulation runs and when the team has capacity to see patients those are met needs. When capacity is less than need, visits expire and move to being unmet. In the simulation, acute primary care visits have the shortest time to becoming unmet as they are more likely to be seen elsewhere (e.g., emergency) or be self-limited (e.g., some infections).

**Fig. 1:**
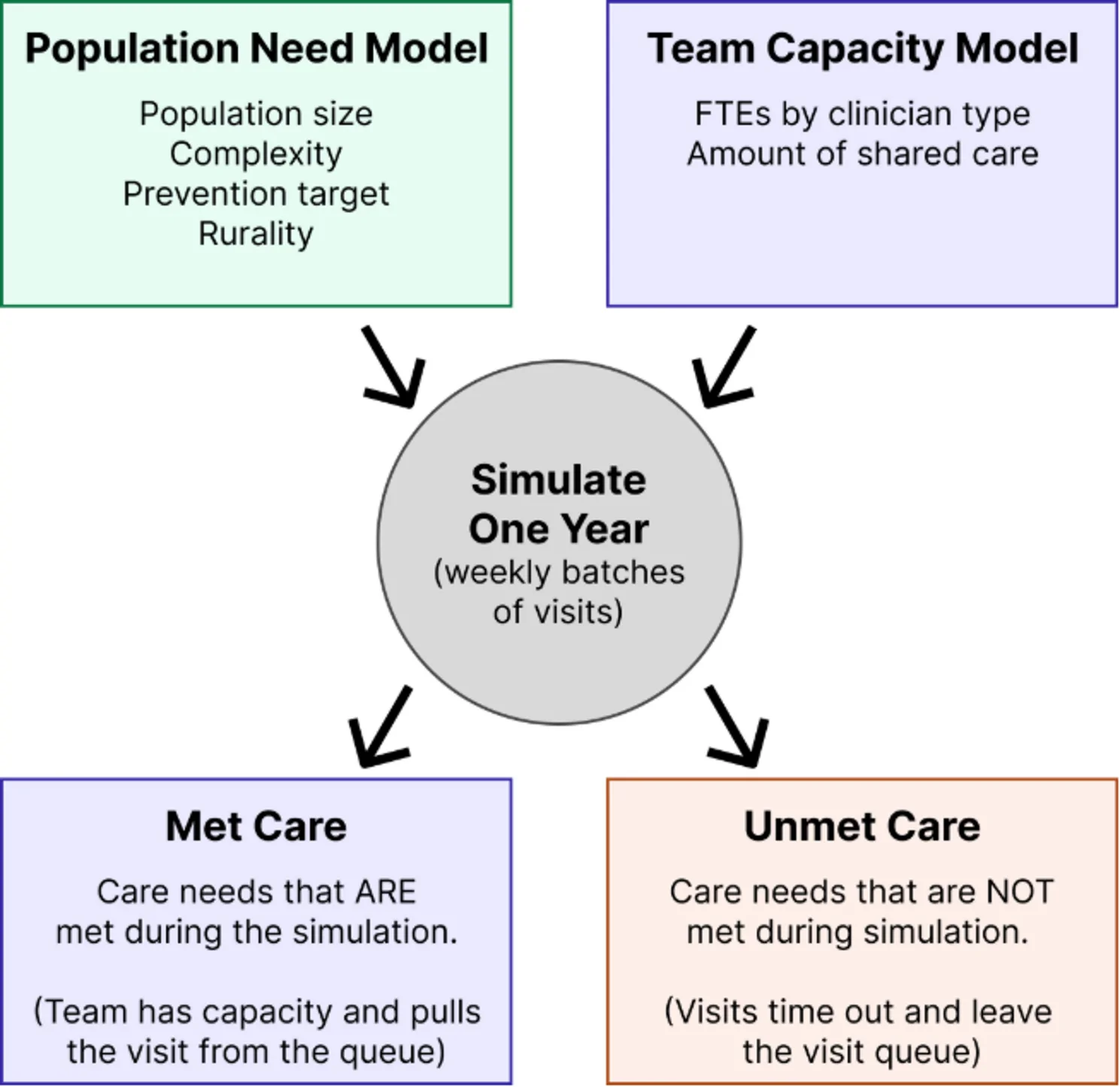
Basic flow of the CapEs simulation. Visit need (predicted weekly) for a population is compared to the visit capacity of the team. As patient visits are “seen” by the team, they are considered met. Those that expire in the queue are considered unmet. Low unmet need is considered a higher capacity match

This is illustrated conceptually in Fig. 1. For more details on the model, see Appendix 1 including Figure S1. The model is designed so that all visit defaults can be customized to match policy targets.

The current version of CapEs is freely available at https://github.com/drpriceless/capes.

### Adding health professions to the team: Delphi results integration

As CapEs v2 was used, planners requested the ability to adjust FTEs per-profession. Because there was insufficient published evidence to include additional health professions, we paused development and completed 7 modified Delphi studies, one for each of the following health professions: nurse, pharmacist, social worker, physiotherapist, occupational therapist, dietitian, and physician assistant (unpublished companion paper, under review). Delphi results (number of primary care visits per day, the percentage of care that could be shared with that profession (as acute, chronic, and preventive primary care)), served as input into CapEs v3 so that the visit capacity and degree of delegation are adjusted by the specific FTEs of the team.

### CapEs user interface (UI) (v3)

**Fig. 2:**
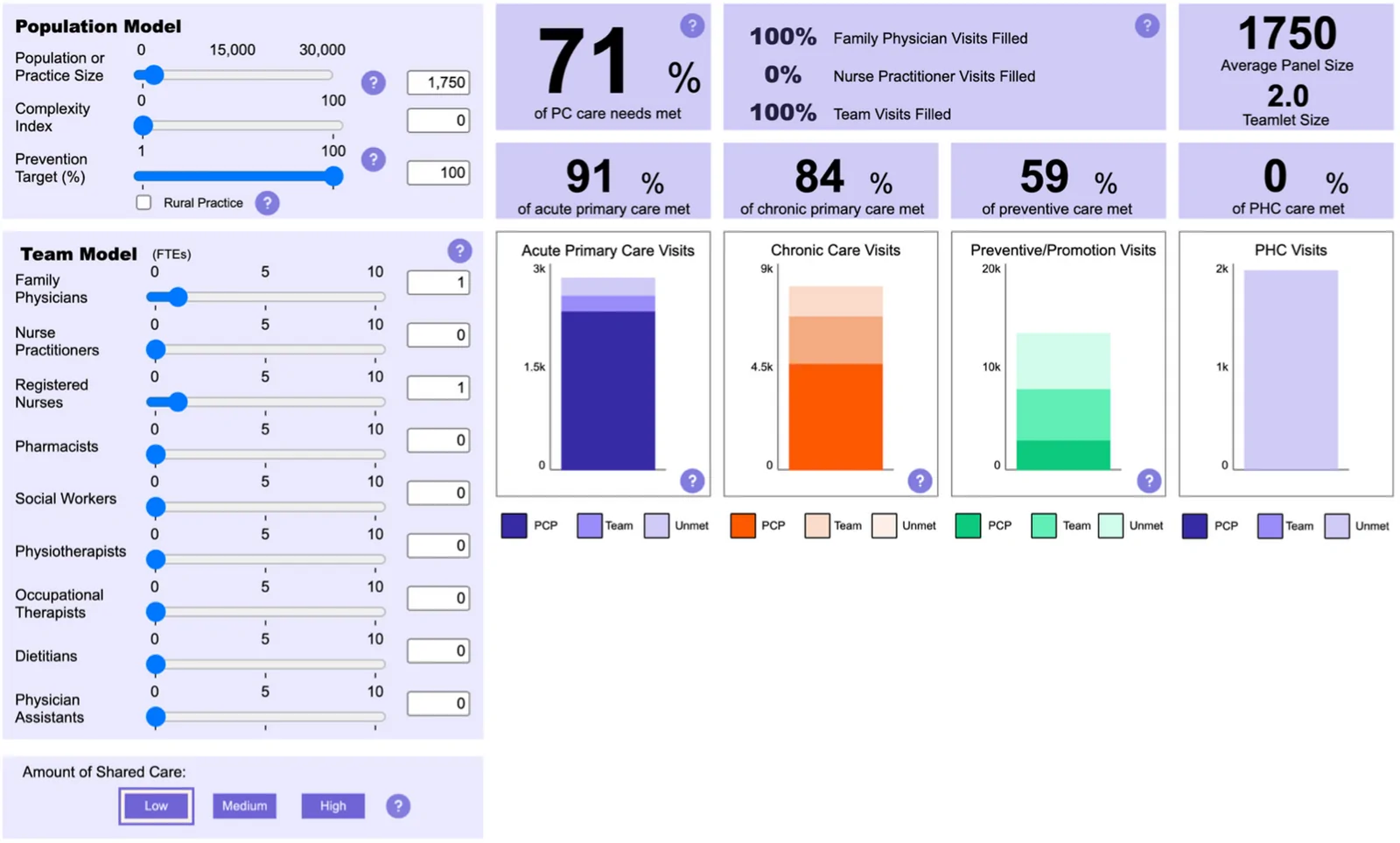
CapEs user interface (v3) showing controls for population model (top left), the team model (side left) and the results (right) that includes needs met and clinician visit utilization. NOTE: Population characteristics (e.g. population size) and FTE counts can go above the slider ranges

Figure 2 shows the CapEs UI. The figure shows a population of 1750 people, low clinical complexity, non-rural. This simple team consists of 1 FTE family physician (FP) and 1 FTE registered nurse with a low amount of shared care (simulating a newer team). CapEs estimates 71% of the care needs are met in this configuration and you can see the breakdown of how the two providers meet the differing needs of acute primary care, chronic care, and preventive care in the graphs in the middle. CapEs also calculates visit utilization for FPs and nurse practitioners (which are modelled as PCPs), other team members, as well as an average panel size.

## Formative evaluation: feedback and benchmarking of CapEs

CapEs has been used with health system planners in reviewing primary care community plans and clinic proposals through facilitated CapEs workshops led by a member of the research team (MP) who is a clinician, researcher, and educator. The workshop allowed for interpretation and application in CapEs of the different data available and an opportunity to discuss implications in a semi-structured manner. Forty-five community plans, clinic proposals, or community data sets have been reviewed in three Canadian provinces (BC, Ontario, New Brunswick), often comparing current state to various future state configurations based on proposal submissions or policy. The workshop format has allowed for discussion with participants to provide context and refine assumptions. The workshops were also helpful in navigating different datasets, as different jurisdictions, communities, or teams had different data to draw from, requiring interpretation and adapting for model parameters. Complexity is challenging to estimate for health planners, particularly when data sources were different or not available.

CapEs allowed planners to reason differently about workforce plans. As an example, in one workshop, the planners initially were only considering how to address an identified population of people who did not have a primary care provider (i.e., unattached) and wanted to see if the plan’s proposed RN FTE could be reduced. When we modelled the proposed team for the unattached patients, CapEs estimated well over 90% of care needs would be met and not all RN visits would be filled. However, in the workshop, we also modelled the full community (including those who were attached and all existing FTEs) and found a significant overall capacity gap. This resulted in discussions on how to distribute the capacity across the community instead of discussing how much to reduce the numbers of RN FTE.

CapEs was used to simulate practices based on the BC policy panel targets (Table 1) to look at the capacity match by calculating the percentage of care met, which is the proportion of the population’s predicted annual visit need that the modeled team meets over a simulated year. The CapEs estimates (Table 3) predict some differences: more care needs may be met in rural communities with smaller panels compared to either urban or vulnerable population practices; however, rural FPs often spend more time in clinical activities outside of clinic (e.g. emergency room and other hospital care) than their urban counterparts [55, 56] suggesting planners are inherently considering this, although there is need to better understand where clinical time is spent to accurately quantify. CapEs predicts a relative increase in care needs met when an RN is added, even when the population targets are adjusted as per the policy. These may indicate either an adjustment is needed in the policy or that the simulation needs to adjust the relative weighting for rural/remote populations and for nurses.

**Table 3:** Comparison of BC panel size targets with CapEs predictions for percent of care needs met for each panel size. For where a range was specified in the policy, the mid-point is provided here

Provider Type	Urban		Rural/Remote		Vulnerable Population	
	BC MoH Panel Target	CapEs % Care Met	BC MoH Panel Target	CapEs % Care Met	BC MoH Panel Target	CapEs % Care Met
Family Physician	1250	61%	800	76%	725	65%
Nurse Practitioner	1000	64%	800	66%	650	62%
FP + Registered Nurse*	1750	71%	1200	79%	Not defined	--

## Discussion

CapEs was developed by the University of British Columbia’s Innovation Support Unit (ISU) at the request of and in collaboration with the BC Ministry of Health, to support them in applying more evidence to PCW planning. It builds on existing work estimating time needed to treat [48, 49] and incorporates it into a simulation model with single screen user interface that allows a user to adjust parameters in real-time. CapEs (model and workshop) operationalizes evidence about primary care time requirements and team capacity into a form that planners can use in local workforce decisions.

In BC, the implementation of CapEs with health planners supported reviewing both community plans and clinic proposals through a workshop. CapEs workshops provide health planners with a different view into primary care capacity to complement the various rules already being used to allocate nursing and allied health clinicians to populations. CapEs provides a complementary exploration of capacity to support reasoning about primary care need and the comparable capacity of teams. CapEs estimates a gap in capacity (as unmet care needs) that is not seen through metrics like attachment. The authors are not aware of a global target for primary care capacity. When thinking about the challenges measuring quality in primary care as a complex adaptive system [57], estimating capacity to help explain outcomes may be useful. Further, setting a 100% target is likely neither feasible nor desirable as it could lead to excess capacity in the system. CapEs also supports PCW planners in considering resource allocation decisions during times of increased fiscal restraint or potential lack of availability. When considering system costs alongside overall patient care needs, it has been helpful for planners to see the predicted impacts of roles on primary care. ISU has run workshops in Ontario and New Brunswick, as part of similar PCW planning activities, looking at 24 additional communities through CapEs.

## Lessons learned

### Data limitations required a simple, adaptable model

Standardized, high-quality local data for primary care workforce planning model are scarce in Canadian jurisdictions. CapEs was kept simple with higher-level levers like “complexity” that could be adjusted based on interpretation of available data and collectively arriving at approximations with planners. This still allowed for PCW planning with more nuance than simple attachment targets.

### Workshop provided needed space for interpretation

Embedding CapEs in a facilitated workshop ensured that interpretations of model parameters were consistent and understood by planners. It also created an educational opportunity for newer health planners. Discussions covered care flows, patient needs, the scope of different primary care professions, and reasonable care goals for different communities. For example, health planners may count funded FTEs, but clinical capacity requires considering only in-clinic time — excluding emergency room coverage and travel to remote communities.

### Capacity match offers a different lens than attachment

Predicting capacity match was often a new concept and proved valuable in workshops. Capacity match, even as an approximation, is more nuanced than a binary attached/unattached metric. Rural communities can have high attachment rates (the percentage of people who are assigned to a regular provider) and lower capacity scores because they typically have larger panels of patients. Capacity can also be estimated for unattached patients served through walk-in clinics — relevant for nearly 1 in 5 Canadians [10].

## Limitations

CapEs has been designed and implemented incrementally, and the current population model is simple. While it provides accessibility (less data is needed about a population or panel), it reduces modeling accuracy. A more accurate approach would embed patient characteristics from the actual population. We have a study underway to look at “visit multipliers” based on population level data to better predict primary care visits/usage/care needs such as age, sex, multimorbidity, socioeconomic status, rurality, and frailty and these data will be considered for future CapEs versions.

A grounded discussion of the scope of primary care is needed. Given the capacity limitations in Canada and elsewhere, difficult conversations may be needed to focus on higher value, evidence based primary care to thus support the greatest number of Canadians. Further, care activities may be better accomplished by other parts of the healthcare system, such as public health programs or community services such as increased scope in pharmacies or even automated systems with reminders and follow up that occurs outside primary care (e.g., for cancer screening).

The CapEs prediction model needs further validation. By using real practice data, we could measure documented rates of adherence to guidelines and compare to capacity estimations from the model between practices. We could assess visit patterns, access measures (e.g., third next available appointment) over time when teams change. Then, on the theoretical side, targets can be created by reviewing best-evidence, guidelines, and policies, calculating time and visit needs similar to Porter [49] and others and using those to inform how scope of primary care changes could also impact capacity.

## Future work

Validation of the CapEs model is needed and is planned as next steps to this work. A validation comparing CapEs predictions to an analysis visits related to common acute, chronic, and preventive care activities would provide greater and more detailed empirical evidence to the model. Also, we are expanding the population model to include patient characteristics, as mentioned earlier. This work is ongoing in collaboration with the Canadian Primary Care Sentinel Surveillance Network (cpcssn.ca), leveraging their data infrastructure and validated case definitions. CapEs’s parameters are localizable, so it could adapt to other health systems, but the visit-model foundations are North American, so other jurisdictions would need local parameterization and validation. Lastly, CapEs did not model digital care. Virtual care (video, phone) has different capacity implications and would be important to incorporate into the model in the future.

## Conclusions

The primary care Capacity Estimator, CapEs, was developed in collaboration with the Ministry of Health of British Columbia. CapEs has been embedded in workshops for PCW planners to help them review workforce plans and options. CapEs has been used with planners in three Canadian provinces at multiple levels of planning (community, clinic, regions). Work is ongoing to extend and better validate CapEs.

Given the ongoing shortage of primary care capacity, tools like CapEs are needed to support more robust primary care workforce planning. This will better address the needs of Canadians who are both the attached and unattached to primary care.

## Supplementary Information

Below is the link to the electronic supplementary material.

Supplementary Material 1

## Data Availability

The current version of CapEs is freely available at https://github.com/drpriceless/capes.

## Abbreviations

CapEs: Capacity Estimator
FTE: Full Time Equivalent
BC: British Columbia
PCW: Primary Care Workforce
UBC: University of British Columbia

## References

[1] Rifkin SB. Alma Ata after 40 years: primary health care and health for all—from consensus to complexity. BMJ Glob Health. 2018;3(Suppl 3). 10.1136/bmjgh-2018-001188. PubMed PMID: 30622747. PMC6307566 30622747

[2] Starfield B, Shi L, Macinko J. Contribution of Primary Care to Health Systems and Health. Milbank Q. 2005;83(3):457–502. 10.1111/j.1468-0009. 2005.00409.x PubMed PMID: 16202000; PubMed Central PMCID: PMC2690145. PMC2690145 16202000

[3] Shi L. The Impact of Primary Care: A Focused Review. Scientifica. 2012;2012:432892. 10.6064/2012/432892. PubMed PMID: 24278694; PubMed Central PMCID: PMC3820521. PMC3820521 24278694

[4] World Health Organization. The World Health Report 2008: primary health care - now more than ever [Internet]. World Health Organization. 2008. Available from: https://iris.who.int/handle/10665/43949.

[5] Duong D. Primary care is facing a capacity crisis - can pandemic lessons help chart a path forward? Can Med Assoc J. 2022;194(43):E1488–1488. 10.1503/cmaj.1096023. PubMed PMID: 36343948. PMC9828995 36343948

[6] Flood CM, Thomas B, McGibbon E. Canada’s primary care crisis: Federal government response. Healthc Manage Forum. 2023;36(5):327–32. 10.1177/08404704231183863. PMC10448296 37424188

[7] Correia T, Kuhlmann E, Lotta G, Beja A, Morais R, Zapata T, et al. Turning the global health and care workforce crisis into action: The pathway to effective evidence-based policy and implementation. Int J Health Plann Manage. 2025;40(1):224–33. 10.1002/hpm.3860. 39396240

[8] Health Canada. Caring for Canadians: Canada’s future health workforce [Internet]. 2025 Jan [cited 2026 Feb 20]. Available from: https://www.canada.ca/en/health-canada/services/health-care-system/health-human-resources/workforce-education-training-distribution-study.html.

[9] Primary Care Needs OurCare. The final report of the largest pan-Canadian conversation about primary care. Toronto, Canada: MAP Centre for Urban Health Solutions; 2024.

[10] Kiran T. Keeping the front door open: ensuring access to primary care for all in Canada. Can Med Assoc J. 2022;194(48):E1655–6. 10.1503/cmaj.221563. PubMed PMID: 36511860. PMC9828980 36511860

[11] Breton M, Green M, Kreindler S, Sutherland J, Jbilou J, Wong ST, et al. A comparative analysis of centralized waiting lists for patients without a primary care provider implemented in six Canadian provinces: study protocol. BMC Health Serv Res. 2017;17(1):60. 10.1186/s12913-017-2007-8. PubMed PMID: 28109279; PubMed Central PMCID: PMC5251310. PMC5251310 28109279

[12] Canadian Institute for Health Information. Supply, Distribution and Migration of Physicians in Canada, 2023 — Data Tables [Internet], Ottawa ON. 2024. Available from: https://www.cihi.ca/en/physicians#:~:text=The%20average%20age%20of%20physicians,males%20versus%2046.4%20for%20females

[13] Premji K, Green ME, Glazier RH, Khan S, Schultz SE, Mathews M, et al. Characteristics of patients attached to near-retirement family physicians: a population-based serial cross-sectional study in Ontario, Canada. BMJ Open. 2023;13(12):e074120. 10.1136/bmjopen-2023-074120. PMC10711930 38149429

[14] Glazier RH. Addressing unmet need for primary care in Canada. Healthc Manage Forum. 2024;37(6):451–6. 10.1177/08404704241271141. 39120449

[15] Statistics Canada. Canada’s population is growing at the fastest pace in the G7 and ranks seventh in the G20 [Internet]. 2022 [cited 2026 Feb 20]. Available from: https://www150.statcan.gc.ca/n1/daily-quotidien/220209/cg-a001-eng.htm.

[16] Lane S, AAPA [Internet]. More patients… More complex patients. 2017 Feb 7 [cited 2026 Feb 20]. Available from: https://www.aapa.org/news-central/2017/02/patients-complex-patients/.

[17] Nolte E, McKee M. Caring for People with Chronic Conditions: A Health System Perspective. McGraw-Hill Education (UK); 2008. p. 288.

[18] Koné Pefoyo AJ, Bronskill SE, Gruneir A, Calzavara A, Thavorn K, Petrosyan Y, et al. The increasing burden and complexity of multimorbidity. BMC Public Health. 2015;15(1):415. 10.1186/s12889-015-1733-2. PMC4415224 25903064

[19] Canadian Medical Association [Internet]. 2022 [cited 2026 Feb 20]. Physician burnout nearly doubles during pandemic. Available from: https://www.cma.ca/about-us/what-we-do/press-room/physician-burnout-nearly-doubles-during-pandemic.

[20] Brown-Shreves D, Wright V, Kiran T. Healthy Debate [Internet]. 2023 [cited 2026 Feb 20]. Team-based care key to alleviating primary-care crisis. Available from: https://healthydebate.ca/2023/04/topic/team-based-care-primary-care-crisis/.

[21] Newton C. It is time to invest in team-based care. Can Fam Physician. 2023;69(2):143–143. 10.46747/cfp.6902143. PubMed PMID: 36813517. PMC9945887 36813517

[22] Alberta Health. Primary Health Care Evaluation Framework [Internet], Government of Alberta. 2013. Available from: https://open.alberta.ca/dataset/9c4dcb17-0b7d-4532-b7c5-9384f02de506/resource/cda5e72c-b9d6-4178-8884-c05619136a3b/download/6862113-2013-primary-health-care-evaluation-framework.pdf

[23] Barbazza E, Kringos D, Kruse I, Klazinga NS, Tello JE. Creating performance intelligence for primary health care strengthening in Europe. BMC Health Serv Res. 2019;19(1):1006. 10.1186/s12913-019-4853-z. PMC6935208 31881884

[24] Bodenheimer T, Ghorob A, Willard-Grace R, Grumbach K. The 10 building blocks of high-performing primary care. Ann Fam Med. 2014;12(2):166–71. 10.1370/afm.1616. PubMed PMID: 24615313; PubMed Central PMCID: PMC3948764. PMC3948764 24615313

[25] Tello J, Barbazza E, Yelgezekova Z, Kruse I, Klazinga N, Kringos D, Indicator Passports. WHO European Primary Health Care, Impact, Performance and Capacity Tool (PHC-IMPACT): version 1 [Internet]. World Health Organization. Regional Office for Europe; 2019. p. 131. Available from: https://iris.who.int/handle/10665/346478.

[26] Sinsky CA, Bodenheimer T. Powering-Up Primary Care Teams: Advanced Team Care With In-Room Support. Ann Fam Med. 2019;17(4):367–71. 10.1370/afm.2422. PubMed PMID: 31285215; PubMed Central PMCID: PMC6827653. PMC6827653 31285215

[27] Bodenheimer T. Building powerful primary care teams. Mayo Clin Proc. 2019;94(7):1135–7. 10.1016/j.mayocp.2019.05.017. PubMed PMID: 31272561. 31272561

[28] Li M, Tang H, Liu X. Primary care team and its association with quality of care for people with multimorbidity: a systematic review. BMC Prim Care. 2023;24(1):20. 10.1186/s12875-023-01968-z. PMC9850572 36653754

[29] Hoff T, Prout K. Physician Use of Health Care Teams for Improving Quality in Primary Care. Qual Manag Health Care. 2019;28(3):121–9. 10.1097/QMH.0000000000000216. 31246774

[30] Hopkins KD, Sinsky C. Taking Team-Based Care to the Next Level. Fam Pract Manag. 2022;29(3):25–31. 35536298

[31] Reid RJ, Coleman K, Johnson EA, Fishman PA, Hsu C, Soman MP, et al. The Group Health Medical Home At Year Two: Cost Savings, Higher Patient Satisfaction, And Less Burnout For Providers. Health Aff (Millwood). 2010;29(5):835–43. 10.1377/hlthaff.2010.0158. 20439869

[32] Sinsky CA, Willard-Grace R, Schutzbank AM, Sinsky TA, Margolius D, Bodenheimer T. Search of Joy in Practice: A Report of 23 High-Functioning Primary Care Practices. Ann Fam Med. 2013;11(3):272–8. 10.1370/afm.1531. PubMed PMID: 23690328; PubMed Central PMCID: PMC3659145. PMC3659145 23690328

[33] Szafran O, Kennett SL, Bell NR, Green L. Patients’ perceptions of team-based care in family practice: access, benefits and team roles. J Prim Health Care. 2018;10(3):248–57. 10. 1071/HC18018 PubMed PMID: 31039939. 10.1071/HC18018 31039939

[34] Lavergne MR, Gonzalez A, Ahuja MA, Hedden L, McCracken R. The relationship between gender, parenthood and practice intentions among family medicine residents: cross-sectional analysis of national Canadian survey data. Hum Resour Health. 2019;17(1):67. 10.1186/s12960-019-0396-y. PMC6694610 31416444

[35] Lavergne MR, Goldsmith LJ, Grudniewicz A, Rudoler D, Marshall EG, Ahuja M, et al. Practice patterns among early-career primary care (ECPC) physicians and workforce planning implications: protocol for a mixed methods study. BMJ Open. 2019;9(9):e030477. 10.1136/bmjopen-2019-030477. PubMed PMID: 31551384; PubMed Central PMCID: PMC6773300. PMC6773300 31551384

[36] Aggarwal M, Hutchison B, Abdelhalim R, Baker GR. Building High-Performing Primary Care Systems: After a Decade of Policy Change, Is Canada Walking the Talk? Milbank Q. 2023;101(4):1139–90. 10.1111/1468-0009.12674. PMC10726918 37743824

[37] Team-Based Care. Health Quality BC [Internet]. 2024 Feb 21 [cited 2024 Feb 14]. Available from: https://healthqualitybc.ca/improve-care/team-based-care/.

[38] Transforming our primary care system through primary care networks [Internet]. British Columbia Ministry of Health. 2019. Available from: https://www2.gov.bc.ca/assets/gov/health/about-bc-s-health-care-system/heath-care-partners/health-newsletter/context-pcn-december-2019.pdf.

[39] Somé NH, Devlin RA, Mehta N, Zaric GS, Sarma S. Team-based primary care practice and physician’s services: Evidence from Family Health Teams in Ontario, Canada. Soc Sci Med. 2020;264:113310. 10.1016/j.socscimed.2020.113310. 32877846

[40] Ozen A, Balasubramanian H. The impact of case mix on timely access to appointments in a primary care group practice. Health Care Manag Sci. 2013;16(2):101–18. 10.1007/s10729-012-9214-y. 23076360

[41] Mwabu G. The Demand for Health Care. Int Encycl Public Health. 2008;84–9. 10.1016/B978-012373960-5.00164-7. PubMed PMID: null; PubMed Central PMCID: PMC7149721.

[42] Simkin S, Chamberland-Rowe C, Bourgeault IL. An integrated primary care workforce planning toolkit at the regional level (part 2): quantitative tools compiled for decision-makers in Toronto, Canada. Hum Resour Health. 2021;19(1):86. 10.1186/s12960-021-00595-y. PMC8293525 34284800

[43] Tomblin Murphy G, MacKenzie A, Alder R, Birch S, Kephart G, O’Brien-Pallas L. An Applied Simulation Model for Estimating the Supply of and Requirements for Registered Nurses Based on Population Health Needs. Policy Polit Nurs Pract. 2009;10(4):240–51. 10.1177/1527154409358777. 20164064

[44] Paige NM, Apaydin EA, Goldhaber-Fiebert JD, Mak S, Miake-Lye IM, Begashaw MM, et al. What Is the Optimal Primary Care Panel Size? A Systematic Review. Ann Intern Med. 2020;172(3):195. 10.7326/M19-2491. 31958814

[45] PCN Service Plan Template. PCN Community Toolkit [Internet]. [cited 2025 Jun 14]. Available from: https://www.pcnbc.ca/en/pcn/permalink/pcn140.

[46] Seixas BV, Regier DA, Bryan S, Mitton C. Describing practices of priority setting and resource allocation in publicly funded health care systems of high-income countries. BMC Health Serv Res. 2021;21(1):90. 10.1186/s12913-021-06078-z. PMC7839200 33499854

[47] Jankowski P, Brown B. Health Care Accessibility Modeling: Effects of Change in Spatial Representation of Demand for Primary Health Care Services. Quaest Geogr. 2014;33(3):39–53. 10.2478/quageo-2013-0028.

[48] Altschuler J, Margolius D, Bodenheimer T, Grumbach K. Estimating a Reasonable Patient Panel Size for Primary Care Physicians With Team-Based Task Delegation. Ann Fam Med. 2012;10(5):396–400. 10.1370/afm.1400. PMC3438206 22966102

[49] Porter J, Boyd C, Skandari MR, Laiteerapong N. Revisiting the Time Needed to Provide Adult Primary Care. J Gen Intern Med. 2023;38(1):147–55. 10.1007/s11606-022-07707-x. PMC9848034 35776372

[50] Muggah E, Graves E, Bennett C, Manuel DG. The impact of multiple chronic diseases on ambulatory care use; a population based study in Ontario, Canada. BMC Health Serv Res. 2012;12(1). Located at: Crossref. 10.1186/1472-6963-12-452. PMC3532841 23217125

[51] Lavergne MR, Bodner A, Peterson S, Wiedmeyer M, Rudoler D, Spencer S, et al. Do changes in primary care service use over time differ by neighbourhood income? Population-based longitudinal study in British Columbia, Canada. Int J Equity Health. 2022;21(1):80. 10.1186/s12939-022-01679-4. PMC9175477 35672744

[52] Lavergne R, Peterson S, Rudoler D, Scott I, McCracken R, Mitra G, et al. Productivity Decline or Administrative Avalanche? Examining Factors That Shape Changing Workloads in Primary Care. Healthc Policy Textbar Polit St E. 2023;19(1):114–29. 10.12927/hcpol.2023.27152. PMC10519339 37695712

[53] Niederberger M, Spranger J. Delphi Technique in Health Sciences: A Map. Front Public Health. 2020;8:457. 10.3389/fpubh.2020.00457. PMC7536299 33072683

[54] Aviva. How to use even over statements for prioritization. Parabol [Internet]. 2020 Oct 29 [cited 2025 Jun 14]. Available from: https://www.parabol.co/blog/strategic-prioritization-even-over-statements/.

[55] Nasim U, Morgan ZJ, Peterson LE. The Declining Scope of Practice of Family Physicians Is Limited to Urban Areas. J Rural Health. 2021;37(4):734–44. 10.1111/jrh.12540. 33244807

[56] Wong E, Stewart M. Predicting the scope of practice of family physicians. Can Fam Physician. 2010;56(6):e219–25. PubMed PMID: 20547504. PMC2902392 20547504

[57] Young RA, Roberts RG, Holden RJ. The Challenges of Measuring, Improving, and Reporting Quality in Primary Care. Ann Fam Med. 2017;15(2):175–82. 10.1370/afm.2014. PubMed PMID: 28289120. PMC5348238 28289120

