# Supplementary material for: CapEs: designing and implementing a Capacity Estimator tool to support primary care workforce planners in Canada
Source: BMC Health Serv Res. 2026 Aug 7;26:1528. doi: 10.1186/s12913-026-15311-6 (PMC13733221; doi:10.1186/s12913-026-15311-6)
Supplement: Supplementary file 1 [file 12913_2026_15311_MOESM1_ESM.docx]

Version: 1.05	Date: 26-June-2026

# CapEs: Designing A Capacity Estimator Tool to Support Primary Care Workforce Planners in Canada.

# Appendix 1: CapEs Model Description

This appendix describes in more detail the CapEs model. The current system dynamics model for CapEs (v3) is divided into three key areas (Figure S1):

Figure S1: Stock Flow diagram of CapEs from Stella Architect. The main sections are colour coded: green is population primary care need, purple is primary care capacity, orange is unmet need, or outflows. Boxes represent stocks (counting accumulated visits); valved arrows are flows that move visits between stocks; circles are converters (describing the factors / parameters in the model, their relationships and contain calculations). Dotted line converters are clones of their original converter (to avoid lines being drawn across the diagram). Items that look like stacks represent arrays (e.g., an array of provider types). Orange arrows visualize the connections between converters. For example (in green) the model has 2 types of variation: Random Variation (week to week perturbations in need) and Seasonal Variation (modeling increases in the fall / winter, and a decrease in summer). These combine to form Total Variation that is then used in the calculation of Weekly Primary Care Need (in visits) shown as a flow that fills up the Primary Care Need stock.

1. **The Population Model** (green) calculates the visit need for the population. It uses the following criteria:

- **Population size**: Number of people in a population or panel. NOTE: numbers can be entered that go above the slider limits.
- **Baseline Visit Need**: Annual average visit need (per person) is based on care time requirements from Porter(49), converted to a visit estimate:
  - **Acute Primary Care**: 0.69 visits / year
  - **Chronic Care**: 2.2 visits / year
  - **Preventive Care**: 4.4 visits / year
  - **Primary Health Care**: 0.69 visits / year. This was an additional category added in later versions of CapEs to begin to illustrate the additional scope and quality brought to primary care by team members beyond the Primary Care Provider (PCP).
- **Population Model Complexity**: Percent of clinical complexity in the population, this translates to increasing the predicted acute and chronic care demands of the population.
- **Prevention Target** (0-100%): This changes the preventive care visit rate. CapEs can simulate Walk-in or episodic only practices (prevention target = 0%), fully longitudinal care (100%) or various combinations.
- **Rurality**: A flag to simulate an increase in scope of practice (and thus visit need) for rural patients. This increases acute and chronic visit need by 1.5x for the population.

2. **Team Model Features** (purple) calculates the overall visit capacity of the team and sets the scope and prioritization of visits for selection. It also calculates the amount of shared care between PCPs and the rest of the team. It uses the following model settings:

- **Full Time Equivalent (FTE) for each role**: Available in the user interface (UI), this allows setting of how much clinic capacity there is by role for each provider type.
- **Visit capacity for each role**: Each role has a daily visit capacity.
- **Shared Care**: The percent of shareable visits is calculated using (a) baseline rates defined for each role, separated into acute primary care, chronic, and preventive care, (b) an overall level of shared care across the team (to simulate more/less mature teams) where high = 100% of baseline, medium = 75%, low = 50%.
- **Teamlet Size**: calculation of the team size per primary care provider. Teamlet Size = 1.0 is no additional team members per primary care provider. Teamlet Size = 2.0 equals 1 FTE team member (any configuration) per FTE of primary care provider. This was used to estimate the clinical capacity lost for articulation work (coordination, communication between the PCP and the team members), expressed in visit equivalents / day of clinical capacity reduced for articulation work.

3. **Unmet Need or Outflows** (orange): As the simulation runs, the primary care need stock (green box) is filled each week, with adjustments for random and seasonal fluctuations. Visits are pulled by provider types in order, based on their role’s priority preference (e.g., acute>chronic>prevention) and shared care rates. Remaining visits have a chance of being unmet and leaving the primary care need stock to “Unmet Needs”. In the simulation, acute primary care visits have both the highest priority for providers and the shortest time to becoming unmet.

The model is designed so that visit defaults can be customized to match policy targets.
